# Different Angles, Changing Perspectives 

**DOI:** 10.3201/eid2604.AC2604

**Published:** 2020-04

**Authors:** Byron Breedlove, Reginald Tucker

**Affiliations:** Centers for Disease Control and Prevention, Atlanta, Georgia, USA

**Keywords:** art science connection, emerging infectious diseases, art and medicine, about the cover, Justinian I, Mosaic of Justinianus I, different angles, changing perspectives, plague, bubonic plague, Yersinia pestis, bacteria, rats, fleas, vector-borne infections, zoonoses

**Figure:**
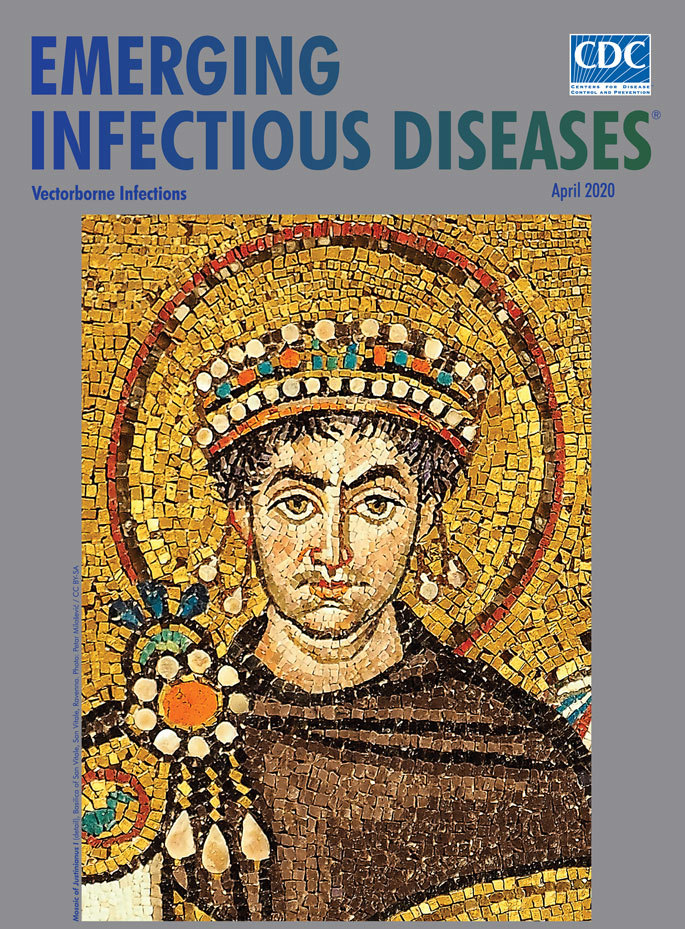
**Artist Unknown. Mosaic of Justinianus I (detail)*,*** Basilica of San Vitale, San Vitale, Ravenna, Italy (ca. 547 A.D.) Photo: Petar Milošević / CC BY-SA

This month’s cover image is a detail from the mosaic of the Emperor Justinian and his court in the Basilica of San Vitale in Ravenna, Italy. Ancient Roman mosaics such as these, typically created by unknown artisans, may be found in private villas and public buildings and provide durable, vivid documentation of ancient Roman life. According to the Getty Museum, many of these intricate, detailed works served as floors in numerous villas and were “designed to be viewed from different angles and to change as your perspective moves.”

The artisans who assembled these mosaics combined thousands of mostly square tiles made from limestone, marble, glass, ceramic, and sometimes precious stones. They arranged these tiles like a complex jigsaw puzzle and affixed them into position with mortar. 

This particular mosaic, viewed as a whole (Figure), depicts the emperor in a ceremonial purple robe with a golden halo, a traditional rendering that symbolizes the importance of the Roman emperor in the Christian church and sets him apart from the more plainly dressed figures surrounding him, further emphasizing the authority of the emperor and his reign. The soldiers to his right and clergy on his left affirm his stature as the center of church and state. The mosaic, which imparts no sense of motion or depth, most likely documents a ceremonial gathering or formal event, perhaps in the same manner that a modern “photo op” might. 

**Figure:**
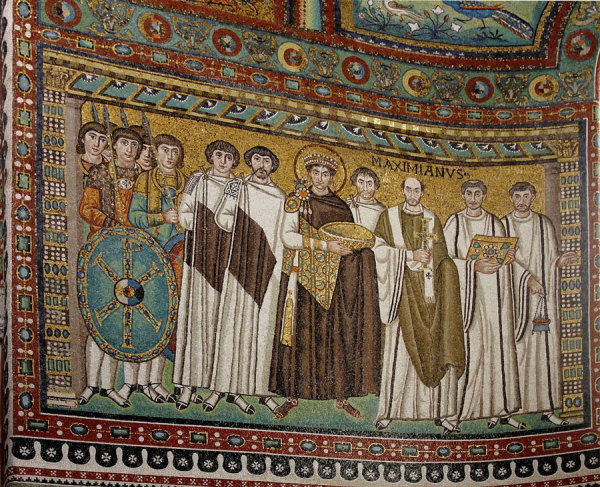
Artist Unknown. Mosaic of Justinianus I, Basilica of San Vitale, San Vitale, Ravenna, Italy (ca. 547 A.D.) Photo: José Luiz Bernardes Ribeiro / CC BY-SA 4.0

Justinian saw himself as the “defender of the faith,” with a mandate to spread that faith throughout the empire. That power, however, did not allow him to escape what historians have called the Plague of Justinian, an outbreak now thought to be due to *Yersinia pestis*, that left him at the brink of death for several weeks, though he did survive. In modern times, scientific progress has enabled clinicians to diagnose suspected cases of plague sooner and administer life-saving treatments with antimicrobial drugs. 
